# Tumor Necrosis Factor-Alpha Polymorphism at Position -238 in Preeclampsia

**DOI:** 10.5812/ircmj.11195

**Published:** 2014-01-05

**Authors:** Mohammad Naderi, Hanieh Yaghootkar, Fatemeh Tara, Jalil Tavakkol Afshari, Reza Farid Hosseini, Majid Ghayour Mobarhan, Abbas Shapouri Moghadam, Masoumeh Mirteimouri, Seyedeh Maryam Tara

**Affiliations:** 1 Mashhad University of Medical Sciences, Mashhad, IR Iran; 2 Genetics of Complex Traits, University of Exeter, Heavitree Rd, Exeter, United Kingdom; 3 Women Health Research Center, Department of Obstetrics and Gynecology, Faculty of Medicine, Mashhad University of Medical Sciences, Mashhad, IR Iran; 4 Immunology Research Center, Department of Immunogenetics, Mashhad University of Medical Sciences, Mashhad, IR Iran; 5 Cardiovascular Research Centre, Avicenna (Bu-Ali) Research Institute, Mashhad University of Medical Sciences, Mashhad, IR Iran; 6 Faculty of Medicine, Shahid Beheshti University of Medical Sciences, Tehran, IR Iran

**Keywords:** Tumor Necrosis Factor-Alpha, Pre-Eclampsia, Polymorphism, Genetic

## Abstract

**Background:**

Preeclampsia is the most common serious disorder during pregnancy and studies show several immune-related processes in its pathophysiology. The role of cytokines and their expression remains controversial in this field. One of the cytokines of interest in recent studies has been TNF-α, which has been shown to have a higher level in maternal plasma of preeclamptic women.

**Objectives:**

This study was designed to evaluate the role of TNF-α polymorphism at position -238 in the risk of developing preeclampsia during pregnancy.

**Patients and Methods:**

One hundred fifty three preeclamptic cases and 140 healthy pregnant women were retrieved from two major hospitals of Mashhad, Iran. Methods a case-control study were designed. Anyone with a history of inflammatory disease, hypertension, or chronic kidney disease was excluded. DNA was extracted from peripheral blood leukocytes. Both groups were genotyped for the polymorphism of the TNF-α gene at position -238 by the RFLP method with Ava II enzyme. Allele and genotype frequencies were compared using one-way ANOVA and the Fisher’s exact test.

**Results:**

There were significant differences between the two groups in TNF-α genotype at position -238 (P < 0.001). In the preeclamptic group, the frequency of the AA genotype was higher (P < 0.001) and the frequency of the GG genotype was lower (P < 0.001). The overall prevalence of the A allele at position -238 was higher in preeclamptic cases (P < 0.001).

**Conclusion:**

In this study group, TNF-α -238 polymorphism was shown to be different in preeclamptic and non-preeclamptic pregnant women. The AA genotype and the A allele may carry an increased risk for developing of preeclampsia.

## 1. Background

Preeclampsia is the most common serious disorder during pregnancy and having a prevalence of 2-5 percent, it leads to significant maternal and neonatal morbidity and mortality (1). Although the main etiology of the disease is still unknown, vast research, has led to a significantly improved understanding of the pathophysiology of preeclampsia and several processes have been suggested to take part. It is believed that in a normal pregnancy there is a condition of controlled mild maternal systemic inflammation, during which circulating levels of particular pro-inflammatory cytokines, such as tumor necrosis factor-α, IL-6, and IL-1 are raised. Maternal peripheral blood leukocytes and trophoblasts appear to be the source of these pro-inflammatory cytokines. Recent studies reveal that immune maladaptation and overt activation of the maternal innate immune system may take part in the pathogenesis of preeclampsia (2).

TNF- α is a T helper-1 cytokine produced by lymphocytes, macrophages and trophoblasts during pregnancy. It functions in several processes that have been found to take part in pathogenesis of preeclampsia, including abnormal placental invasion, endothelial cell damage, and oxidative stress (3). In the last two decades, elevated serum levels of TNF-α, and sTNF receptors as well as increased mRNA/protein expression of TNF/TNFRs were noticed in leukocytes and placenta of preeclamptic women (4). Taking into account the inheritable nature of preeclampsia (5, 6) there has been widespread research for identifying any association between a number of genes and the risk for developing preeclampsia, and TNF-α has been a part of these investigations. Several studies have looked for an association between preeclampsia and different single nucleotide polymorphisms in the promoter region of TNF-α gene which could affect its expression. Much work has been done on the -308 polymorphism which a meta-analysis showed no association with preeclampsia (7).

## 2. Objectives

Considering the difference in the prevalence of the TNF-α genotype in different ethnicities (8), we studied the role of TNF-α genotype in preeclampsia by determining the correlation between the -238 polymorphism of the TNF-α gene with preeclampsia in the pregnant women referring to two main hospitals (Qaem and Omolbanin) of Mashhad, the second biggest city of IR Iran.

## 3. Patients and Methods

In a case-control study, 153 preeclamptic cases and 140 healthy pregnant women were collected from two major hospitals (Qaem and Omolbanin) of Mashhad, Iran. Preeclampsia was defined as newly diagnosed blood pressure after the 20th week of gestation accompanied by proteinuria (more than 300 mg protein concentration in the 24hr urine) (9). Hypertension was measured as a systolic pressure over 140 mmHg and/or diastolic pressure over 90 mmHg and gestational age was measured according to the last menstrual period (LMP). All of the participant were otherwise healthy and had no history of inflammatory, hypertensive, or chronic kidney diseases. They were fully informed about the study protocol and gave an informed consent prior to entering the study. The sampling method was non-probability and available. A 10 cc blood sample was obtained from each case at study initiation and transferred to lab tubes containing 5% EDTA. The DNA was extracted from the leukocytes by the salting out method (10) and the purity of the final product was assessed using spectrophotometry.

A 71 base pair sequence of TNF-α promoter was amplified by PCR using the device Biometra- T3-Thermoblock model. A primer sequence of 20 nucleotides was designed by the Gene Runner software and checked with the Gene Bank and EMBL (European Molecular and Biology Library). The Primer sequence was as follows: 

TNF-α (-238) Forward: 5´ GGT CCT ACA CAC AAA TCA GT 3´

TNF-α (-238) Reverse: 5´ CAC TCC CCA TCC TCC CTG GTC 3´

The PCR cycles included a primary 3-min cycle at 94˚C, 43 cycles of three parts (1 min at 94˚C, 1 min at 60˚C, and 1 min at 72˚C), and a final 5-min cycle at 72˚C. The PCR product underwent RFLP and electrophoresis and was dyed by silver nitrate where the sliced parts were clarified by applying the Ava II enzyme. Afterwards, the -238 TNF-α genotype was analyzed. The entire studied variables for both groups were collected and recorded in special forms.

Sample size was calculated using frequency data of previous studies with a power of 80% and a minimum detectable odds ratio of 2.5. A p-value of less than 0.01 was considered significant. The demographic and laboratory obtained data were analyzed by the SPSS software (version 11.5). One-way ANOVA was used for comparing frequencies between the three genotype groups. Allele and genotype frequencies were compared using a 2x2 contingency table with Fisher’s exact test. T-test was applied on quantitative variables with normal distribution (age and blood pressure) and Chi-square test was used for proportion analysis of qualitative variables (parity and family history of hypertension). The deviation from Hardy-Weinberg equilibrium was also assessed using chi-square test.

## 4. Results

The clinical characteristics of the study population are presented in (Table 1). There was no significant difference between age of cases and controls. In the preeclamptic group, more people had a family history of hypertension (45.8% vs. 23.6%, P < 0.001). There was no difference in parity between the two groups. Positive family history of preeclampsia in first degree relatives correlated with preeclampsia (P < 0.001). Four individuals from the control group were excluded during the PCR-RFLP phase due to technical issues. The genotypic distribution of -238 TNF- α differed significantly between the two groups (P < 0.0001) (Table 2). The frequency of the AA genotype was higher and the GG genotype was lower in the preeclamptic group (P < 0.001 and P < 0.001, respectively). The frequency of the A Allele was higher in the preeclamptic group than the control group (P < 0.001) (Table 3). The TNF-α -238 genotypes in both the case and control groups were found to be in Hardy-Weinberg equilibrium (P = 0.0002 and P = 0.587 respectively). Data of mean systolic and diastolic blood pressures in various genotypes are shown in (Table 4). The diastolic blood pressure differed significantly between the three genotypes (P < 0.001).

**Table 1.** Clinical Characteristics of Participants

	Preeclamptic	Non-Preeclamptic
**Age, Mean ± SD**	28.2 ± 6.1	27.1 ± 6.3
**Parity, No. (%)**		
Nulliparous	70 (51.9)	64 (45.7)
Multiparous	65 (48.1)	76 (54.3)
**Family History of Hypertension, No. (%) ** ^**a**^	70 (45.8)	32 (22.9)

^a^ P < 0.001

**Table 2.** Genotype Frequency Distribution of -238 TNF-α

Genotype	AA ^a^, No. (%)	GA, No. (%)	GG ^a^, No. (%)	Total, No. (%)
**Case**	27 (90)	104 (72.2)	22 (19.1)	153 (52.9)
**Control**	3 (10)	40 (27.8)	93 (80.9)	136 (47.1)
**Total**	30 (100)	144 (100)	115 (100)	289 (100)

^a^ P < 0.001

**Table 3.** Allelic Frequency of -238 TNF-α

	A^a^	G
**Preeclamptic, No. (%)**	158 (51.6)	148 (48.4)
**Non-preeclamptic, No. (%)**	49 (18)	223 (82)
**Total, No. (%)**	207 (35.8)	371 (64.2)

^a^ P < 0.001

**Table 4.** Systolic and Diastolic Pressures in Different Genotypes

	AA	GA	GG
**Systolic BP, mean ± SD**	149.0 ± 26.7	142.8 ± 22.9	118.8 ± 23.4
**Diastolic BP ^**a**^, mean ± SD**	92.8 ± 12.0	90.4 ± 15.9	73.0 ± 14.3

^a^ P < 0.001

## 5. Discussion

Like other inflammatory diseases with familial and genetic components (6), there has been increasing effort to find the genes that are responsible for increased risk of developing preeclampsia. The candidate parts of the genome are HLA genes (11), as well as genes expressing molecules that studies have suggested to have a role in pathogenesis; including molecules in hemostasis (e.g. thrombomoduline), molecules in hemodynamics (e.g. Angiotensin and renin), molecules affecting endothelial function (e.g. eNOS), Inflammatory cytokines (e.g. TNF-α), and some other molecules which take part in response to oxidative stress, lipid metabolism, angiogenesis and endocrine processes (12).

The idea of TNF-α having a part in the process has been initiated and supported by several findings. A meta-analysis of 24 studies has shown that the level of TNF-α in the maternal plasma is higher in preeclamptic women (13). However there has been no difference in placental tissue levels and TNF-α expression between preeclamptic and normal placenta (14-17); a finding that may point to the maternal origin of higher TNF-α. Several in Vitro and animal studies also support and clarify the role of TNF-α in preeclampsia. In vitro studies have shown that TNF-α has an apoptotic impact on cultured trophoblasts (18), (19, 20) and has a negative effect on syncitiation (21). In an animal study on pregnant rats, reductions in uterine perfusion pressure (RUPP) led to an increase in plasma TNF-α levels and induction of hypertension. In the same study administration of soluble TNF-α receptor antagonist, etanercept, blunted the hypertension (22).

Genetic studies have been conducted on polymorphisms at different locations in the promoter region of TNF-α, that could possibly have an effect on its level of expression. Single nucleotide polymorphisms at positions -850 (1, 23, 24), -308 (25), and -238 (3) have been investigated. While several studies found a significant correlation between a specific genotype at position -308 TNF-α and susceptibility to preeclampsia, a meta-analysis of 16 studies found none (26). In this study, we found an association between polymorphisms at -238 TNF-α and the risk of developing preeclampsia in the Iranian population. Preeclampsia had a positive correlation with the AA genotype and the A allele, and a negative correlation with GG genotype and the G allele. Another study conducted in Iran, Tehran, displayed opposite results, with the GG genotype and the G allele being associated with preeclampsia (3). However, in that study, all of the 100 people in the control group had the GA genotype, and among the 160 people in the preeclamptic group, none had the AA genotype, which may be a result of selection bias or the small number of participants.

Our study faced some limitations. The significant cost of laboratory tests limited the number of participants and lowered the power, making the negative results unreliable. We also couldn’t make a random sampling as a result of limited available cases. Our study, according to its type (case-control), was prone to some bias concerning the history of patients. Our data lacked a baseline protein excretion and blood pressure level to assist in making the diagnosis of preeclampsia. We recommend conducting a larger study with sufficient participants and more power on different polymorphisms of TNF-α and other theoretically supported molecules.
